# A Rare Soft Tissue Tumor Masquerading as a Parathyroid Adenoma in a Patient with Birt-Hogg-Dubé Syndrome and Multiple Cervical Endocrinopathies

**DOI:** 10.1155/2014/753694

**Published:** 2014-12-25

**Authors:** Kael V. Mikesell, Afif N. Kulaylat, Keri J. Donaldson, Brian D. Saunders, Henry S. Crist

**Affiliations:** ^1^Department of Pathology, Penn State Milton S. Hershey Medical Center, Hershey, PA 17033, USA; ^2^Department of Surgery, Penn State Milton S. Hershey Medical Center, Hershey, PA 17033, USA

## Abstract

Birt-Hogg-Dubé (BHD) syndrome is an autosomal dominant disorder that presents with renal tumors, pulmonary cysts with spontaneous pneumothoraces, and skin hamartomas. We present a case of a 67-year-old female with multiple endocrinopathies and a history of BHD syndrome. In 2011, a thyroidectomy with a four-gland parathyroidectomy was performed for toxic multinodular goiter (TMNG) and parathyroid hyperplasia. On frozen section, a tumor was identified next to a hypercellular parathyroid. After being worked up, this tumor was determined to be an adult rhabdomyoma. This represents the first time that both TMNG and parathyroid hyperplasia have been present in a BHD patient. Additionally, this is the first adult rhabdomyoma reported in a patient with BHD syndrome. Adult rhabdomyomas have no reported associations; however, potential colocation of the mutation in BHD syndrome and translocation in adult rhabdomyomas on chromosome 17p suggests a possible connection. Further work is needed to better understand this connection.

## 1. Introduction

**Figure 1:**
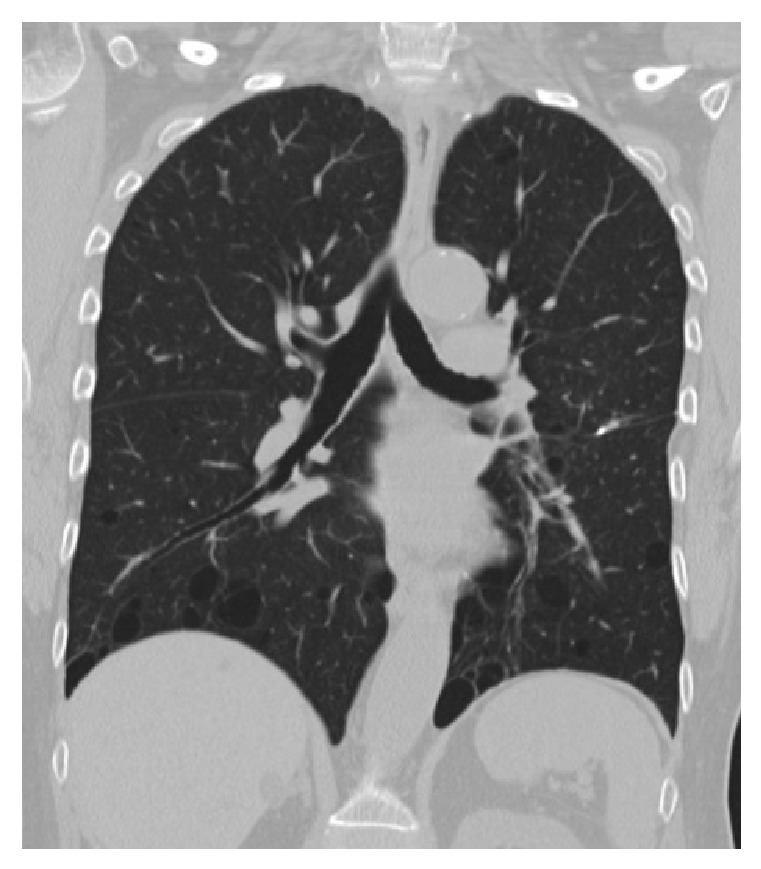
Coronal section of chest CT scan demonstrating numerous pulmonary cysts preferentially located in the lower lobes.

Birt-Hogg-Dubé (BHD) syndrome is a rare autosomal dominant disorder that is widely variable in its clinical expression. It is caused by a germline mutation in the tumor suppressor gene folliculin (FLCN) [1]. The syndrome was first described by Birt et al. in 1977 in reference to a triad of benign skin tumors [2, 3]. These skin tumors generally occur on the face and neck and consist of fibrofolliculomas and trichodiscomas, hamartomas made up of the pilar apparatus's ectodermal and mesodermal components [4]. Acrochordona also may be seen near these hamartomas [5]. Since its first description, additional clinical signs have been added to the BHD syndrome. Pulmonary cysts (preferentially located in the lower lobes and the perimediastinal area) and pneumothoraces are generally the presenting symptom for BHD syndrome (Figure 1) [6]. Renal tumors (the most important marker of prognosis in patients with BHD syndrome [4]) may also occur with chromophobe renal cell carcinoma (RCC) making up the majority of these tumors [7]. Other renal tumors, including oncocytomas and hybrid oncocytic chromophobic tumors, are also common [8].

Although these are the currently recognized clinical findings in BHD syndrome, other potential associations have been proposed in the literature [2, 3, 9–11]. We present a rare tumor initially presumed to be a parathyroid adenoma in the setting of a BHD syndrome patient with primary hyperparathyroidism and toxic multinodular goiter.

## 2. Case Presentation

**Figure 2:**
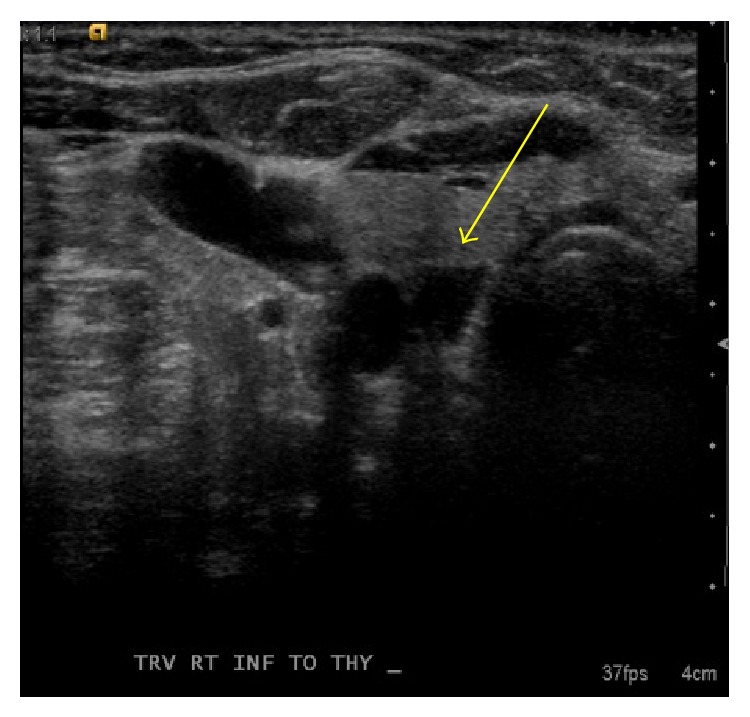
Hypoechoic mass (0.5 cm × 0.6 cm) medial to the carotid artery and lateral to the trachea suggestive of parotid adenoma; arrow points to hypoechoic mass.

We present a 67-year-old female with a history significant for two episodes of spontaneous pneumothoraces and findings of numerous pulmonary cysts on computer tomography (CT) scan, with incidental discovery of thyroid nodules (Figure 1). Following biochemical evaluation, she was diagnosed with subclinical hyperthyroidism and hypercalcemia. Her thyroid stimulating hormone (TSH) was suppressed at 0.09 (0.45–4.50 mIU/mL), with normal levels of free T3 and T4. Her calcium was elevated at 10.6 mg/dL (8.4–10.2 mg/dL), as was her intact parathyroid hormone (PTH) at 191 pg/mL (9–73 pg/mL). She had a concomitant decrease in her phosphorus level to 2.3 mg/dL (2.5–4.9 mg/dL) and 25-hydroxy vitamin D to 9 ng/mL (30–74 ng/mL) and normal 24-hour urine calcium levels. Bone density scans demonstrated significant central and peripheral osteoporosis along with prior CT scans exhibiting evidence of asymptomatic nephrolithiasis. The combination of these laboratory values in addition to nuclear medicine thyroid uptake scan and cervical ultrasound led to the diagnosis of toxic multinodular goiter (TMNG) and primary hyperparathyroidism. Cervical ultrasonography suggested right superior parathyroid adenoma (Figure 2).

Following medical and surgical endocrinology evaluation, numerous similar skin lesions were investigated with biopsies consistent with fibrofolliculomas. Subsequent abdominal imaging would reveal a left-sided exophytic renal mass for which she underwent robotic-assisted laparoscopic partial nephrectomy and was found to have a renal oncocytoma. The patient's history was also significant for two parotid oncocytomas resected at age of 52 and 61, respectively. Birt-Hogg-Dubé syndrome was suspected by the clinicians and later confirmed with genetic testing through GeneDx (Gaithersburg, MD) positive for the FLCN gene mutation, specifically IVS4-2 A>G, also referred to as c.250-2 A>G. She had no family history of parotid, thyroid, parathyroid, renal, or colonic tumors.

In June 2011, the patient underwent total thyroidectomy and parathyroid exploration. Two hypercellular parathyroids and one intrathyroidal parathyroid were removed from the patient which brought her baseline parathyroid hormone (PTH) 302 pg/mL to 27 pg/mL (9–73 pg/mL). The left inferior gland was visualized and appeared normal. Nineteen months after removal of the parathyroids, the patient had a calcium level of 9.4 mg/dL (8.4–10.2 mg/dL) and a PTH of 12.5 pg/mL.

**Figure 3:**
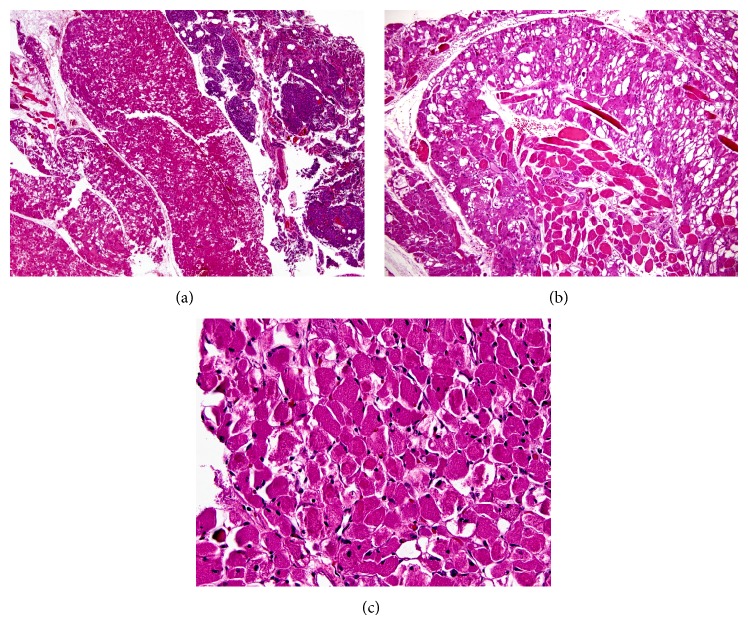
Rhabdomyoma. (a) Low power view of the rhabdomyoma (left) with adjacent hypercellular parathyroid (right). (b) Normal striated muscle intertwined with tumor cells. (c) High power view of rhabdomyoma; notice the eosinophilic granular cytoplasm with small eccentrically and centrally placed nuclei. Much of the cytoplasm is retracted from the cell membrane. (Hematoxylin-eosin, original magnification ×40 (a), original magnification ×100 (b), and original magnification ×400 (c).)

Final pathology of the thyroid gland demonstrated nodular hyperplasia. No C-cell hyperplasia was seen by H&E or by calcitonin immunohistochemical stains. One of the parathyroid specimens labeled “right superior parathyroid gland” was a brown-red fragment of soft tissue that was thought to be a parathyroid adenoma perioperatively and an oncocytoma on frozen section. It measured 1.1 × 0.2 × 0.2 cm and weighed 0.16 grams. Microscopically, the tumor contained large round to rhomboid-shaped cells with a low nuclear to cytoplasmic ratio and both central and eccentric nuclei. The cytoplasm demonstrated an eosinophilic and granular appearance with crystalline intracellular structures. Frequent vacuolation of the cytoplasm was also seen and no mitotic activity was identified. Scattered small skeletal muscle bundles were intertwined within the tumor (Figure 3). A small fragment of hypercellular parathyroid was seen adjacent to the tumor. A differential diagnosis was established which included oncocytoma, rhabdomyoma, hibernoma, or granular cell tumor.

**Figure 4:**
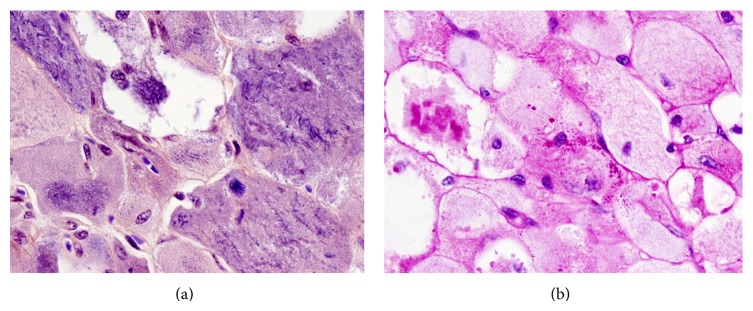
Special stains of the rhabdomyoma. (a) PTAH stain showing characteristic haphazard cross striations and intracytoplasmic crystals. Cross striations and intracytoplasmic crystals are stained blue with the PTAH stain. (b) PAS stain demonstrating positive stain of glycogen granules. The granules are staining eosinophilic and are noted to be intracytoplasmic. (phosphotungstic acid-hematoxylin stain, original magnification ×1000 (a), and Periodic Acid Schiff stain, original magnification ×1000 (b)).

To further work up the case, immunohistochemical and special stains were employed. The tumor cells did show some weak scattered staining for S100 but were negative for myogenin, desmin, smooth muscle actin (SMA), parathyroid hormone (PTH), thyroid transcription factor-1 (TTF-1), and thyroglobulin. On H&E, our suspicion was high for a muscle derived tumor, and even though the tumor cells were negative for immunohistochemical stains for muscle markers, we found that the benign skeletal muscle that was intertwined in the tumor was also negative for the stains (note: during that time in our laboratory, we were having difficulty procuring appropriate staining with the muscle specific immunohistochemical stains). As a result, this led us to rely less heavily on immunohistochemical stains for determining the diagnosis. Phosphotungstic acid-hematoxylin (PTAH) stain demonstrated that the intracellular crystalline structures seen on hematoxylin and eosin stain (H&E) were poorly formed striations (Figure 4(a)). A Periodic Acid Schiff (PAS) stain highlighted intracellular globules within the cytoplasm of the tumor cells (Figure 4(b)).

**Figure 5:**
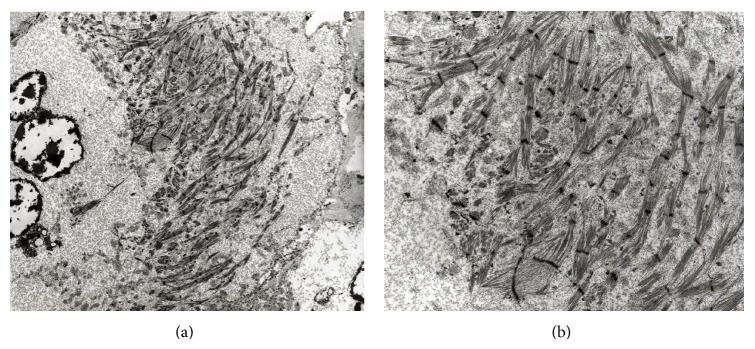
Ultrastructure of the rhabdomyoma. (a) Electron microscopy demonstrating skeletal muscle fibers with Z-bands, extensive glycogen, and a small nucleus. (b) Electron microscopy, higher power, better illustrating the muscle fibers, and surrounding glycogen. (Electron microscopy, original magnification ×2950 (a) and original magnification ×6600 (b).)

Although the tumor did not stain well with immunohistochemical stains for muscle markers, the PTAH and PAS stains demonstrated a typical staining pattern that would be seen in muscle fibers. For confirmation of its derivation from muscle, electron microscopy (EM) on formalin fixed tissue was attempted to evaluate the ultrastructure of the tumor. The EM was successful and demonstrated an extensive amount of glycogen adjacent to and surrounding unorganized myofibrils which contained prominent Z-band formation (Figure 5). A small number of mitochondria were also identified. The EM definitively showed that the tumor was of muscle origin and a final diagnosis of adult rhabdomyoma was rendered.

## 3. Discussion

There are a few interesting points to derive from this case. First, while there have been independent reports of either TMNG or parathyroid adenomas in the setting of BHD syndrome, this is the first case of both TMNG and 4-gland parathyroid hyperplasia reported in the literature in the setting of BHD syndrome [3, 12–14]. Other reported associations with BHD syndrome include medullary thyroid cancer, thyroid adenoma, parotid oncocytoma, lipomas, angiolipomas, intestinal polyposis, colonic adenocarcinoma, and neural tissue tumors [2–4, 10, 11]. No causal relationship between BHD syndrome and these tumors has been proven [4]. Our patient also developed parotid oncocytomas, which in the setting of BHD syndrome has also been reported in three other reports [10, 15, 16]. There are only two other reports of multinodular goiter in association with BHD syndrome [13, 14]. The nature of the association of these conditions with BHD syndrome is incompletely understood.

Second, to the best of our knowledge, this case represents the first adult rhabdomyoma diagnosed in a patient with BHD syndrome. Rhabdomyomas are rare benign mesenchymal neoplasms of skeletal muscle that are classified according to their location and degree of differentiation. Some rhabdomyomas are prevalent in specific syndromes, the most important example being cardiac rhabdomyomas in patients with tuberous sclerosis (TS). TS is an autosomal dominant syndrome that, like BHD syndrome, presents with renal tumors (most commonly angiomyolipomas), lung cysts, and skin hamartomas in addition to other clinical findings. Although TS and BHD are clearly unique syndromes, recent articles demonstrated that both FLCN protein (mutated in BHD syndrome) and tuberous sclerosis complex (TSC) protein (mutated in TS syndrome) lead to activation of mammalian target of rapamycin complex 1 (mTORC1) of the mTOR pathway. mTORC1 reduces autophagy of cells, and its activation leads to an increase in risk for tumors, especially renal tumors [17–19].

Adult rhabdomyomas are rare tumors composed of well differentiated skeletal muscle found most commonly in the head and neck region. They have not been associated with TS syndrome, BHD syndrome, or any other syndrome. As previously mentioned, however, BHD syndrome may have more associations than was originally identified [20, 21]. So, could there be an unrecognized association between BHD syndrome and adult rhabdomyomas? The answer may be found by evaluating the cytogenetics of these entities.

In the early 1990s, karyotyping was performed on an adult rhabdomyoma which demonstrated a reciprocal translocation between chromosome 15q and 17p (46, XY, t(15; 17) [q24; p13]) [22]. Since all of the cell cultures for this research were derived from a single tumor source, the external generalizability of the findings is limited, and a literature search found no additional cytogenetic studies of this tumor. Although adult rhabdomyomas are quite rare, performing cytogenetics in five to ten of these tumors would give us more information about whether this translocation is reproducible. In BHD syndrome, the mutation in the FLCN gene that encodes for folliculin is at 17p11.2 [23]. If the adult rhabdomyoma cytogenetics is reproducible, this suggests that the translocation in adult rhabdomyomas and the mutation in BHD syndrome are possibly colocalized on chromosome 17p. A plausible association between adult rhabdomyomas and BHD syndrome could further be explained by comparative genomic hybridization (CGH) or direct sequencing. Unfortunately, these studies could not be performed on our specimen because FLCN gene germline mutation would be present in all cells including the rhabdomyoma tumor cells. At this point, a possible association is interesting, but purely speculative.

## 4. Conclusion

This case presents an interesting constellation of findings in a patient with BHD syndrome including parotid oncocytomas, toxic multinodular goiter, primary hyperparathyroidism, and a rhabdomyoma. The proximity of the gene mutation in BHD syndrome and translocation in the adult rhabdomyoma could suggest a possible association. Work on BHD syndrome and its possible associations is incomplete. A better understanding of this rare disease will likely occur as further understanding of the mTOR pathway is uncovered.
